# Aflatoxins and Ochratoxin A in Tea Sold in Lebanon: Effects of Type, Packaging, and Origin

**DOI:** 10.3390/ijerph20166556

**Published:** 2023-08-10

**Authors:** Hussein F. Hassan, Hadeel Tashani, Farah Ballouk, Rouaa Daou, André El Khoury, Mohamad G. Abiad, Ali AlKhatib, Mahdi Hassan, Sami El Khatib, Hani Dimassi

**Affiliations:** 1 Nutrition Program, Department of Natural Sciences, School of Arts and Sciences, Lebanese American University, Beirut P.O. Box 13-5053, Lebanonmahdi-hassan2015@hotmail.com (M.H.); 2 Department of Nutrition and Food Sciences, School of Arts and Sciences, Lebanese International University, Beirut P.O. Box 146404, Lebanon; 3 Centre d’Analyses et de Recherche, Unité de Recherche Technologies et Valorisation Agro-Alimentaire, Faculty of Sciences, Campus of Sciences and Technologies, Saint Joseph University of Beirut, Mar Roukoz P.O. Box 17-5208, Lebanon; 4 Department of Nutrition and Food Sciences, Faculty of Agricultural and Food Sciences, American University of Beirut, Beirut P.O. Box 11-0236, Lebanon; 5 Laboratories for the Environment, Agriculture, and Food (LEAF), Faculty of Agricultural and Food Sciences, American University of Beirut, P.O. Box 11-0236, Beirut 1107-2020, Lebanon; 6 Department of Food Sciences and Technology, School of Arts and Sciences, Lebanese International University, Bekaa P.O. Box 146404, Lebanon; sami.khatib@liu.edu.lb; 7 Center for Applied Mathematics and Bioinformatics (CAMB), Gulf University for Science and Technology, P.O. Box 7207, Hawally 32093, Kuwait; 8 School of Pharmacy, Lebanese American University, Byblos P.O. Box 36, Lebanon

**Keywords:** tea, ochratoxin, total aflatoxins, ELISA, contamination

## Abstract

Tea is among the oldest and most-known beverages around the world, and it has many flavors and types. Tea can be easily contaminated in any of its production steps, especially with mycotoxins that are produced particularly in humid and warm environments. This study aims to examine the level of ochratoxin A (OTA) and total aflatoxin (AF) contamination in black and green tea sold in Lebanon, evaluate its safety compared to international standards, and assess the effect of different variables on the levels of OTA and AFs. For this, the Lebanese market was screened and all tea brands (*n* = 37; 24 black and 13 green) were collected twice. The Enzyme-Linked Immunoassay (ELISA) method was used to determine OTA and AFs in the samples. AFs and OTA were detected in 28 (75.7%) and 31 (88.6%) samples, respectively. The average of AFs in the positive (above detection limit: 1.75 μg/kg) samples was 2.66 ± 0.15 μg/kg, while the average of OTA in the positive (above detection limit: 1.6 μg/kg) samples was 3.74 ± 0.72 μg/kg. The mean AFs in black and green tea were 2.65 ± 0.55 and 2.54 ± 0.40 μg/kg, respectively, while for OTA, the mean levels were 3.67 ± 0.96 and 3.46 ± 1.09 μg/kg in black and green tea samples, respectively. Four brands (10.8%) contained total aflatoxin levels above the EU limit (4 μg/kg). As for OTA, all samples had OTA levels below the Chinese limit (5 μg/kg). No significant association (*p* > 0.05) was found between OTA and tea type, level of packaging, country of origin, country of packing, and country of distribution. However, AF contamination was significantly (*p* < 0.05) higher in unpacked tea, and in brands where the country of origin, packing, and distributor was in Asia. The results showed that the tea brands in Lebanon are relatively safe in terms of AFs and OTA.

## 1. Introduction

Mycotoxins are harmful secondary metabolites produced by different fungi, such as *Aspergillus*, *Penicillium*, *Alternaria*, *Fusarium*, and *Claviceps*. Mycotoxins have the potential to contaminate food and severely harm humans [1]. Molds may be found in a wide range of crops and foods, including cereals, nuts, spices, dried fruits, apples, and coffee beans. They thrive mostly in warm and humid circumstances [2]. Mycotoxins can have multiple negative health consequences and offer a major health risk to both humans and animals [2]. The Food and Agriculture Organization (FAO) reported that mycotoxins had contaminated around 25% of the world’s food crops [3]. In general, because of greater humidity and warmth, crops in tropical and subtropical locations are more susceptible to mycotoxin contamination [4]. The carcinogenic, mutagenic, teratogenic, and immunosuppressive properties of mycotoxins can result in major health issues in both humans and animals [5]. 

Aflatoxins are among the most toxic mycotoxins, generated by molds such as *Aspergillus flavus* and *Aspergillus parasiticus,* which thrive in soil, rotting plants, hay, and grains [2]. Cereals, oilseeds, spices, and tree nuts are among the crops that are commonly contaminated by *Aspergillus* species [2]. Moreover, according to the European Food Safety Authority, mycotoxins can be ingested through contaminated food or acquired from animals fed contaminated feed. Aflatoxins and ochratoxin A are the most widespread mycotoxins. Large quantities of aflatoxins can cause acute poisoning (aflatoxicosis) and death usually through liver damage [2]. According to the WHO, aflatoxins have also been found to be genotoxic, and there is evidence that they can cause liver cancer [2]. Total aflatoxins (AFs) are the sum of aflatoxins B1, B2, G1, and G2, and they are well known for their high toxicity and negative consequences [2]. AFs are genotoxic, carcinogenic, teratogenic, mutagenic, hepatotoxic, and immunosuppressive [6]. On the other hand, ochratoxin A (OTA) is a prevalent food-contaminating mycotoxin produced by various *Aspergillus* and *Penicillium* species, and common food items that are contaminated include cereals and cereal goods, coffee beans, dried vine fruits, wine and grape juice, spices, and licorice [2]. Due to its teratogenicity, cancer-causing nature, mutagenicity, hepatotoxicity, genotoxicity, immunotoxicity, embryotoxicity, formative poisonousness, neuro-harmfulness, testicular harmfulness, blood-brain barrier harm, and nephrotoxicity, OTA was designated as a group 2B (possible human carcinogen) for humans, as opposed to aflatoxins, which were classified as a first-group carcinogen for humans [7,8,9,10,11]. 

The tea plant originates from Southeast China, and nowadays is cultivated in more than 50 countries [12]. Traditional *Camellia sinensis* (*C. sinensis*) black and green teas are among the world’s oldest beverages [13,14]. Tea comes in a range of flavors that vary according to the oxidation and fermentation techniques used [15]. According to the FAO, the worldwide tea market has expanded quickly in recent decades due to the increasing consumption of tea beverages [16]. In fact, tea is now the second most consumed beverage after water [17]. Among the six categories produced worldwide, 70% is black tea, 20% is green tea, and oolong tea is less than 2%. Thus, black tea is the most popular category all over the world, accounting for 70% of total tea consumption worldwide [18]. 

Tea is one of the matrices of interest in mycotoxin testing since mycotoxins are common crop pollutants (Duarte et al., 2020). Teas are easily contaminated by fungi during production and processing. For example, *Aspergillus* spp., *Penicillium* spp., *Mucor* spp., *Rhizopus* spp., *Absidia* spp., and *Alternaria* spp. were frequently found during processing [19]. From the atmosphere, phyllosphere, and soil of a tea factory, [20] identified 34 fungal species, including *Aspergillus niger*, *A. flavus*, and *Fusarium lactis*, the species being considered as mycotoxigenic fungi. Tea packaging steps may as well involve fungal contamination. Poor storage and long-term exposure to air can increase the risk of fungal contamination. During transportation, tea might be wet-back, resulting in secondary contamination by toxigenic fungi if it is not appropriately packaged (sealed and sufficient dried) and stored. In other words, poor farming techniques and improper processing, drying, packing, storage, and transportation conditions encourage fungal development, increasing the risk of mold growth. A subtropical environment, which is ideal for tea growing, is also ideal for the formation of toxigenic mold [21]. As a result, contamination with mycotoxins is a critical food safety concern, particularly in Asia [22].

Despite the negative consequences of mycotoxins on public health, only a few studies [23,24,25,26,27,28,29,30] assessed OTA and AFs in tea, no data exist about these mycotoxins in tea sold in Lebanon and the Arab world. Therefore, this study aims to evaluate the safety of the tea sold in Lebanon, in terms of OTA and AF levels, and to assess the effect of different variables on the mycotoxin contamination in tea.

## 2. Materials and Methods

### 2.1. Sample Collection

A market screening was carried out in September 2021 by visiting major supermarkets in Lebanon. A total of 74 samples coming from 37 brands (collected twice) were included in the study. Among the brands collected, 24 (65%) and 13 (35%) of samples were black and green, respectively. On the other hand, 30 (81%) had both primary and secondary packages, while 7 (19%) had only primary packaging. Concerning the country of origin, 15 (41%) and 13 (35%) were from Asia and Europe, respectively, while the country of origin in 9 (24%) samples was not mentioned on the package. Additionally, 15 (41%) and 13 (35%) were packed in Asia and Europe, respectively, while the country of packing was missing from the label in 9 (24%) samples. As for the country of the distributor, 14 (38%) and 14 (38%) were located in Asia and Europe, respectively, while 9 (24%) samples lacked the country of the distributor (Table 1). Each brand was labeled with a unique code. The samples were stored in the freezer (−18 °C). The information present on the labels was used to characterize the tea in terms of brand, type, whether the tea was packaged in primary packaging only or in primary and secondary packaging, the country of origin, country of packing, and country of distributor.

### 2.2. AF and OTA Determination 

Enzyme-Linked Immunoassay (ELISA) was used to measure the AFs and OTA in tea. ELISA is one of the most often used methods for the analysis of mycotoxins. Two samples from each brand, with each sample having different production date, were tested in duplicate. The different steps of the clean-up, extraction, and kit implementation were carried out according to the manufacturer’s protocol. To ensure adequate clean-up, immunoaffinity columns were used prior to the ELISA testing. 

For AFs, clean-up was carried out according to the manufacturer’s instructions. Tea samples were prepared first by adding 5 g of ground tea samples to 25 mL of methanol (70%) then they were extracted by mixing for 10 min at 300 rpm. The extract was then filtered using a paper filter. Afterwards, 15 mL of distilled water were added to 5 mL of the filtered solution along with 0.25 mL of Tween 20 and stirred for 2 min. After that, the samples were passed through the RIDA^®^ Aflatoxin columns (Art. No. R5001/R5002; R-biopharm, Pfungstadt, Germany) as follows. First, the columns were washed with 2 mL distilled water and then filled with 1 mL of the prepared sample solution. The extracts were passed slowly at a flow rate of 1 drop/sec and at a continuous manner using a vacuum unit. Following that, the columns were rinsed with 10 mL distilled water and the air was pressed through the columns to ensure the complete discarding of residues. Finally, the columns were eluted with 0.5 mL of methanol. During the elution, a slow flow rate was maintained to ensure the complete elution of aflatoxins. The collected eluent was then used in the subsequent RIDASCREEN^®^ Aflatoxin Total assay plate.

As for OTA clean-up, 5 g of ground sample were weighed into a 50 mL tube and 25 mL of 75% methanol/0.13 M NaHCO_3_ buffer 50/50 *v/v* were added. The mixture was extracted afterwards by mixing at 300 rpm for 10 min. Following that, the extract was filtered by passing the mixture through a paper filter. A volume of 5 mL of the filtered extract was obtained and 15 mL of phosphate-buffered saline (PBS) buffer was added along with 0.25 mL Tween 20. The mixture was then stirred for 2 min and passed afterwards through RIDA^®^ OTA columns (R1303). First, the columns were rinsed with 10 mL PBS/methanol 90/10 *v/v* and then the entire diluted prepared sample solutions were passed. The extracts were passed slowly at a flow rate of 1 drop/sec and at a continuous manner using a vacuum unit. Following that, the columns were rinsed with 10 mL of PBS/methanol 90/10 *v/v* and air was pressed through the column to ensure the complete discarding of residues. Finally, the column was eluted with 1 mL of methanol (100%). During the elution, a slow flow rate was maintained to ensure the complete elution of ochratoxin. The collected eluents were used afterwards in the RIDASCREEN^®^ Ochratoxin A assay plate. 

A RIDASCREEN^®^ Aflatoxin Total (R4701) (R-biopharm, Pfungstadt, Germany) (detection limit: 1.75 μg/kg) kit was used to determine the AFs. A sufficient number of microtiter wells were inserted into the microwell holder for all the standards and samples. The standard and sample positions were recorded. After that, 50 µL of the standard or prepared sample followed by 50 µL of the conjugate were added. Next, 50 µL of the antibody was added to each well, and the plate was manually shaken gently and incubated for 30 min at room temperature. The liquid was poured out of the wells and the microwell holder was vigorously tapped upside down against absorbent paper to ensure the complete removal of liquid from the wells. All the wells were filled with 250 µL of wash buffer and emptied. The latter step was repeated two more times. Next, 100 µL of substrate/chromogen was added to each well, and the plate was shaken manually and incubated for 15 min at room temperature. Then 100 µL of the stop solution was added to each well, and the plate was shaken manually before measuring the absorbance at 450 nm within 30 min after the addition of the stop solution.

A RIDASCREEN^®^ Ochratoxin A (R1312) (R-biopharm, Germany) (detection limit: 1.6 μg/kg) kit was used to determine the OTA. A sufficient number of microtiter wells was inserted into the microwell holder. Next, 50 µL of the standard solutions or prepared sample was added to separate duplicate wells. Then 20 µL of conjugate was added to each well. The plate was mixed gently by shaking it manually and then incubated for 30 min at room temperature in the dark. The liquid was poured out of the wells and the microwell holder was vigorously tapped upside down against absorbent paper. The wells were filled with 250 µL wash buffer and poured out. The washing procedure was repeated two times. Next 200 µL of substrate/chromogen was added to each well. The plate was shaken manually and incubated for 15 min at room temperature in the dark. Then 100 µL of the stop solution was added to each well. The plate was manually shaken gently and the absorbance was measured at 450 nm and read within 15 min after the addition of the stop solution.

### 2.3. Statistical Analysis

Data were coded and entered into SPSS V27 for analysis. Both concentrations of AFs and OTA above the detection limits were tested for normal distributions. The difference in means between the groups was tested using the independent t-test for two groups and the one-way ANOVA for more than two groups. When the ANOVA test showed statistical significance, post hoc analysis was carried out using the Bonferroni correction for pair-wise comparisons, which corrects the family-wise type I error. A significance level of 0.05 was used.

## 3. Results and Discussion

There are no current maximum limits of mycotoxins in teas and medicinal plant infusions in the European Union. The European Pharmacopoeia established a maximum of 4 μg/kg for all four aflatoxins in medicinal plants [17]. As for OTA, there are no standard limits set by the EU as well; however, some studies utilize the limits set by the Chinese government, which is 5 μg/kg for OTA [31].

In the last couple of decades, mycotoxins and heavy metal contaminations in teas were not investigated much. Although Halt et al. examined the molds and mycotoxins in tea as early as 1998, the studies on mycotoxin contaminations in teas are limited. From the literature available, it is obvious that the prevalence and contamination levels of mycotoxins were inconsistent from study to study. Although regional environmental factors (e.g., climate, temperature, and air humidity) may account for these differences, variations in sampling and determination methods used in the studies might also contribute to the inconsistencies. The methodology studies indicated that the selectivity, accuracy, and sensitivity of the analytical methods could significantly affect the final results [31,32]. 

In this study, AFs and OTA were detected in 28 (75.7%) and 31 (88.6%) samples, respectively. The average of the AFs in positive (above detection limit: 1.75 μg/kg) samples was 2.66 ± 0.15 μg/kg, while the average of OTA in positive (above detection limit: 1.6 μg/kg) samples was 3.74 ± 0.72 μg/kg. Four brands (10.8%) contained total aflatoxin levels above the EU limit (4 μg/kg), while all samples had OTA levels below the Chinese limit (5 μg/kg).

The association between AF and OTA levels in tea and different variables was assessed. These variables include the type of tea (green vs. black), packed in primary and secondary packaging or packed in primary packaging only, the countries of origin and packing, and the country of distributor (located in Asia, Europe, or not known) (Table 2 and Table 3). AF contamination was higher in tea with primary packaging only for both AF and OTA; however, it was significantly (*p* < 0.05) higher in AFs only. This can be due to the fact that when primary and secondary packaging are present, the barrier properties are enhanced, and thus, the moisture content of the tea will not increase during transportation and storage, resulting in the absence of mold growth and subsequent aflatoxin production. In addition, AF contamination was significantly (*p* < 0.05) higher in brands where the country of origin, packing, and distributor is in Asia vs. in Europe. This can be due to the fact that weather conditions in Asia tend to be hot and humid, favoring the mold production and mycotoxin secretion, in addition to the fact that the good agricultural practices, good manufacturing practices, and good storage practices are not well developed, implemented, and maintained in many Asian countries.

Black tea production involves additional steps, such as withering and fermentation to achieve the oxidation of tea polyphenols, and subsequent theaflavin and thearubigin condensation. This may increase the risk of fungal contamination [33]. In addition, among all tea types, dark teas are associated with most of the microorganisms, which play significant roles in developing tea quality and flavors. Thus, it is expected that dark tea is the tea type that is most susceptible to contamination with several mycotoxins. Furthermore, since black teas generally have a complex fungal flora, there is the possibility of being contaminated with mycotoxins [34]. In this study, AFs and OTA were higher in black tea samples, but the different was not significant (*p* > 0.05) (Table 2 and Table 3).

### 3.1. Comparisons with Other Studies and What the Current Work Adds to the Existing Knowledge

This study is the first in Lebanon and the Arab world to assess the prevalence of mycotoxins in tea sold in Lebanon. This is of great importance since tea is one of the main beverages consumed in the eastern part of the world, including Lebanon [35]. In addition, food safety in Lebanon has been at stake in the last decade due to poor control from the inspection authorities [36]. In fact, previous studies conducted in Lebanon mainly investigated the presence of mycotoxins in herbs, spices, rice, wheat, and infant formulas, among others, but not including tea, which is a frequently consumed beverage in Lebanon [37,38,39,40,41,42]. Compared to the aforementioned studies from Lebanon, the prevalence of mycotoxins in tea in this study is lower than what was reported in other food products (human milk, dairy products, rice, and spices). Very few studies in the literature assessed mycotoxins in tea. In Turkey, no total aflatoxin or aflatoxin B1 was determined in 79 tea samples [43]. A recent Iranian study revealed that among 97 tea samples, 36% and 38% reported OTA with a maximum level of 0.51 and 4.7 µg/kg in green and black tea, respectively, which were lower than findings in this study [44]. On the other hand, in Morocco, none of the samples exceeded the OTA limit, which is in line with other results [29]. In Pakistan, [45] reported an AF concentration range in tea between 0.11 and 16.17 µg/kg, which is higher than results in this study. In Latvia, [17] found OTA in 10% of the tea samples and the range was between 2.99 and 30.3 µg/kg, which is higher than what is reported in this study.

According to [34], from the data available in publications to date, a total of 274 green tea samples were screened for mycotoxin contaminations. The main mycotoxins detected from green teas are AFs and OTA. OTA occurrence is 88.9% with the contaminations ranging from 0.01 to 20.35 μg/kg (average 4.842 μg/kg). Ref. [24] reported a mean OTA level of 7.22 μg/kg in green tea and 6.26 μg/kg in black tea, which was higher than what this study reported (3.46 μg/kg and 3.67 μg/kg, respectively). On the other hand, the OTA contamination level in black tea reported in this study (3.67 μg/kg) was comparable to findings of [46] in Turkey (3.02 μg/kg), of [47] in Poland (3.87 μg/kg), and of [48] in Spain (3.85 μg/kg).

It is worth mentioning that no studies in the literature investigated the effect of the type of tea, presence of packaging, countries of origin and packing, and the main distributor on the presence of mycotoxins. Packed tea contained significantly (*p* < 0.05) lower AFs. This is because the packaging protects the tea from mold growth and thus mycotoxin secretion. In addition, whenever the country of origin, country of packing, or the country of the distributor was in Asia, the AFs were significantly (*p* < 0.05) higher. This could be due to poor agricultural, manufacturing, storage, and distribution practices in some Asian countries of [45,49,50]. 

### 3.2. Study Strengths and Limitations

The strengths of this study include the fact that it is the first in Lebanon and the Arab region to evaluate the safety of tea sold in Lebanon in the country in terms of total aflatoxins and OTA content. In addition, this study analyzed tea in packs, as in general, the residents of Lebanon purchase packed tea. 

With regard to the limitations, the results contained some outliers. This could be attributed to a chance of false positive or negative results due to an insufficient blockage of the surface of the microtiter plate immobilized with antigen, and future experiments should be carried out in triplicate to solve this. In addition, future studies must include tea without any packaging as tea is sold as such in some areas of Lebanon, especially rural ones, making it more prone to fungal growth and contamination with mycotoxins. Furthermore, the exposure to mycotoxins from tea beverage consumption must be assessed to reveal the magnitude of this hazard. In addition, the effect of different tea preparations must be evaluated as different areas in Lebanon prepare the beverage differently. Finally, comparing the results from ELISA technique must be validated with HPLC before using the reported values in calculating the exposure assessment to mycotoxins from tea consumption in Lebanon. 

## 4. Conclusions

Tea is one of the oldest and most-known beverages in the world, and it can be easily contaminated with mycotoxins at any step of its production. In this study, four brands (10.8%) contained total aflatoxin levels above the limit (4 μg/kg), while all samples had OTA levels below the limit (5 μg/kg). No significant association (*p* > 0.05) was reported between OTA and tea type, the presence of packaging, the country of origin, the country of packing, and the country of distribution. On the other hand, AF contamination was significantly (*p* < 0.05) higher in tea with primary packaging only, and in brands where the country of origin, packing, and distribution was in Asia. The results from this study showed that the tea brands in Lebanon are relatively safe in terms of AFs and OTA. On the other hand, being a commonly consumed beverage in Lebanon, more attention must be given to the safety of tea sold in the country. More research and monitoring studies are needed to analyze the multi-mycotoxin incidence in tea. In addition, the official limits of mycotoxins in tea must be developed and monitored by the local authorities. Future studies should investigate other contaminants, such as toxic metals, and assess the effect of preparation method on the mycotoxin levels in the beverage itself. 

## Figures and Tables

**Table 1:** Characteristics of tea samples (*n* = 37).

	*n* * (%)
**Type**	
Black	24 (65)
Green	13 (35)
**Packing**	
Packed in primary and secondary packages	30 (81)
Packed in primary package only	7 (19)
**Country of Origin**	
Europe	8 (22)
Asia	15 (41)
Not known	14 (38)
**Country of Packaging**	
Europe	13 (35)
Asia	15 (41)
Not known	9 (24)
**Main Distributer**	
Europe	14 (38)
Asia	14 (38)
Not known	9 (24)

* n: number.

**Table 2:** Effect of the different variables on total aflatoxins.

	Mean ± SD *	*p*-Value
**Type**		
Black	2.65 ± 0.55	
Green	2.54 ± 0.40	0.649
**Packing**		
Packed in primary and secondary packages	2.85 ± 0.45	
Packed in primary package only	3.27 ± 0.42	**0.041**
**Country of Origin**		
Europe	2.67 ± 0.36	
Asia	3.04 ± 0.48	
Not known	2.79 ± 0.42	**0.032**
**Country of Packaging**		
Europe	2.62 ± 0.35	
Asia	2.99 ± 0.46	
Not known	3.12 ± 0.45	**0.012**
**Main Distributer**		
Europe	2.64 ± 0.37	
Asia	2.87 ± 0.40	
Not known	3.35 ± 0.43	**<0.001**

* SD: standard deviation.

**Table 3:** Effect of the different variables on OTA.

	Mean ± SD *	*p*-Value
**Type**		
Black	3.67 ± 0.96	
Green	3.46 ± 1.09	0.852
**Packing**		
Packed in primary and secondary packages	3.32 ± 1.07	
Packed in primary package only	3.68 ± 0.38	0.768
**Country of Origin**		
Europe	3.63 ± 0.95	
Asia	3.45 ± 0.49	
Not known	3.99 ± 0.28	0.669
**Country of Packaging**		
Europe	3.62 ± 1.08	
Asia	3.19 ± 1.10	
Not known	3.08 ± 0.56	0.735
**Main Distributor**		
Europe	3.67 ± 0.94	
Asia	3.08 ± 0.59	
Not known	3.25 ± 0.54	0.613

* SD: standard deviation.

## Data Availability

Available upon request.
